# Comprehensive study of dexamethasone on albumin biogenesis during normal and pathological renal conditions

**DOI:** 10.1080/13880209.2020.1855214

**Published:** 2020-12-17

**Authors:** Qin Gong, Jilei Yin, Mulan Wang, Luling He, Fan Lei, Yingying Luo, Shilin Yang, Yulin Feng, Jun Li, Lijun Du

**Affiliations:** a School of Pharmacy, Jiangxi University of Traditional Chinese Medicine, Nanchang, China; b Pharmacology Laboratory, State Key Laboratory of Innovative Drugs and Efficient Energy-Saving Pharmaceutical Equipment, Nanchang, China; c Department of Traditional Chinese Medicine, Jiangsu Union Technical Institute Lianyungang Branch Institute of Traditional Chinese Medicine, Lianyungang, China; d School of Life Sciences, Tsinghua University, Beijing, China

**Keywords:** Cisplatin, acute kidney injury, proteinuria, gene transcription start site

## Abstract

**Context:**

Dexamethasone (DXM) has an anti-immunoinflammatory effect, and is often used in acute kidney injury (AKI). However, the effects of DXM on albumin (ALB) have not been fully studied.

**Objective:**

To investigate the effects of DXM on ALB production and renal function.

**Materials and methods:**

Male Wistar rats were divided into normal and DXM groups (0.25, 0.5, 1 mg/kg for 5 days) (*n* = 15) for a dose-dependent study. Rats were divided into normal group and DXM groups (0.5 mg/kg for 3, 5, 7 days) (*n* = 9) for a time-dependent study. In AKI experiment, rats were divided into normal (saline), cisplatin (CP, 5 mg/kg, i.v.), CP + DXM groups (0.25, 0.5 and 1 mg/kg, i.m.) (*n* = 16). The blood and the organs were isolated for analysis.

**Results:**

In normal, serum ALB (sALB) and serum total protein (sTP) increased in DXM group with sALB increased 19.8–32.2% (from small to large dosages); and 30.2–32.5.6% (from 3 to 7 days of DXM); sTP 15.7–22.6% and 14.2–24.3%; urine ALB (uALB) 31.5–392.3%, and 1047.2–1390.8%; urine TP (uTP) 0.68–173.1% and 98.0–504.9%, compared with normal groups. DXM increased the mRNA expression of *Cebp* and *Hnf*, suppressing podocin. In AKI, DXM decreased serum BUN (53.7%), serum Cre (73.4%), sALB (30.0%), sTP (18.7%), uALB (74.5%), uTP (449.3%), rescuing the suppressed podocin in kidney.

**Conclusions:**

DXM acts on *Cebp* and *Hnf* and promotes ALB production. This finding helps to evaluate the rationale of DXM for kidney injury.

## Introduction

Dexamethasone is a corticosteroid drug with anti-inflammatory and immunosuppressive effects that are associated with NFκB/IL-1β signalling, as well as the NLRP3 inflammasome (Guan et al. 2020). Dexamethasone is a commonly used drug for clinical immunoinflammatory therapy, such as for the treatment of acute kidney injury, liver injury, and inflammatory injury (Takahira et al. 2001; Ryan et al. 2020). DXM reduces pain and inflammation and plays an anaesthetic role in peripheral nerve block (Short et al. 2020; Tammachote and Kanitnate 2020). Accumulating evidence has shown that DXM has a significant effect on apoptosis and autophagy inhibiting or promoting these processes in a dose-dependent manner (Xue et al. 2016; Wang et al. 2018; Zhang et al. 2018). Generally, high doses of DXM can induce apoptosis and inhibit autophagy (Zhang et al. 2007; Zeng et al. 2017; Deng et al. 2019), while low doses can inhibit apoptosis and induce autophagy (Fong et al. 2007; Laane et al. 2009; Yin et al. 2010; Tsai et al. 2015; Kosutova et al. 2016; Liu et al. 2018). In addition, DXM inhibits cell proliferation, arresting cells in the G0/G1 phase, which is accompanied by apoptosis (He et al. 2016; Li et al. 2018). DXM is the ligand of the glucocorticoid receptor (GR), acting on GR and phosphorylating the receptor, which translocates into the nucleus, promoting the expression of genes as a transcription factor and inducing effects (Marchetti et al. 2003; Ibrahim et al. 2018; Dey and Bishayi 2019; Murani et al. 2019).

During kidney injury, a decrease in serum protein levels and an increase in urine protein levels occur (Gong, He, Wang, Ouyang, et al. 2019), suggesting that the permeability of the glomerular basement membrane is increased. Thus, monitoring protein concentration and preventing serum protein levels from being too low is one of the important components in the treatment of kidney injury. DXM can prevent and treat renal damage, exert anti-inflammatory effects, and inhibit the apoptosis of glomerular podocytes. (Wada et al. 2005; Yu and Li 2013). During the treatment of renal damage, DXM was shown to increase serum ALB and total protein levels by promoting the expression and synthesis of ALB in liver cells (Schwartz et al. 1982; Jefferson et al. 1985; Yeoh et al. 1985). However, the underlying mechanism by which DXM upregulates ALB expression increases serum protein levels and influences renal function has not yet been reported.

In our previous study, we observed that DXM could significantly increase serum protein levels in normal rats, as well as increase urine protein levels. In addition, DXM increases both serum and urine proteins in animals with acute kidney injury due to various pathological causes. Proteinuria reflects an increase in the permeability of the glomerular basement membrane. Clinically, high sALB is closely related to the occurrence of acute kidney injury (Thongprayoon et al. 2018) and eventually causes chronic kidney damage by inducing an inflammatory response (Carney 2018) because high concentrations of ALB can cause damage to renal tubular epithelial cells and glomerular podocytes (Simpson and Shand 1983; Jarad et al. 2016; Xiao et al. 2016). Thus, urinary ALB excretion is often used as an indicator of acute kidney injury (Bolisetty and Agarwal 2011). The underlying mechanism by which DXM induces both sALB and uALB increase is worth studying in-depth.

We hypothesized that DXM could promote ALB transcription and that high levels of serum ALB might hurt podocytes in the glomerular basement membrane and influence renal function. These findings will help to comprehensively evaluate the rationale of the use of DXM as an anti-inflammatory immune drug to treat kidney injury and answer the question of how to better apply DXM to avoid adverse reactions. As such, we conducted in-depth and systematic research on the pharmacological effects of DXM on normal rats and cisplatin-induced acute kidney injury rats.

## Materials and methods

### Animals

Male Wistar rats (170 ± 10 g) were purchased from Hunan SJA Laboratory Animal Co., Ltd. [SCXK (Xiang) 2016-0002]. This study was completed in the Laboratory of Barrier Environment of Jiangxi Bencao-Tiangong Technology Co., Ltd. [SYXK (Gan) 2018-0002]. The animals were housed in temperature- and humidity-controlled rooms under a 12 h light/dark cycle and provided unrestricted amounts of rodent chow and water. All procedures described were reviewed and approved by the Institutional Animal Care & Use Committee of Jiangxi University of Traditional Chinese Medicine (TCM) and the Animal Welfare & Ethics Committee of Jiangxi University of TCM (approval ID: 1901-JunLi-B4). The experimental procedures strictly followed the Experimental Animal Welfare and Ethics Guidelines of China.

### Chemical and materials

Injectable dexamethasone was purchased from Anhui Golden Sun Biochemical Pharmaceutical Co., Ltd. (batch number: 15032521) (Anhui, China). Injectable cisplatin was purchased from Jiangsu Hansoh Pharmacy (batch number: 180804) (Lianyungang, China). Urea nitrogen (BUN) (R1 TG836, R2 TG837), total protein (TP) (TH619) and albumin (ALB) (TF126) kits were all purchased from Nippon Chun Yao Pharmaceutical Co., Ltd. (Shanghai, China). Total urine protein (CSF) (706061E) and creatinine (Cre) (710241H) kits were purchased from Beijing Leadman Biochemistry Co., Ltd. (Beijing, China). The urinary microalbumin (mALB) (batch number: 0118051) kit was purchased from Makerbio Biochemical Co., Ltd. (Chengdu, China).

### Experimental procedures

#### Dose-dependent analysis of DXM

Male Wistar rats were randomly divided into four groups (*n* = 15): the normal group and three groups treated with 0.25, 0.5 and 1 mg/kg DXM by intramuscular injection (i.m.) for 5 days. On day five, nine rats were killed with anaesthesia, blood was collected, and the liver and kidney were isolated for analysis. The rest of the rats were observed continuously for 15 days without any treatments. Finally, blood was collected, and the kidneys and livers were isolated for examination.

#### Time-dependent analysis of DXM

Male Wistar rats were randomly divided into four groups (*n* = 9): the normal group and three groups treated for 3, 5 and 7 days with 0.5 mg/kg DXM administered by intramuscular injection. On days 3, 5, and 7, blood was collected, and the livers and kidneys of six rats were isolated for analysis. The rest of the rats were observed continuously for 15 days without any treatments. Then, the rats were sacrificed under anaesthesia, and blood was collected for examination.

#### Effect of DXM on acute kidney injury

Male Wistar rats were divided into five groups (*n* = 16): the normal group, the CP group (5 mg/kg cisplatin, intravenous administration), and the CP + DXM groups (0.25, 0.5 and 1 mg/kg DXM, intramuscular injection). After CP injection, DXM was administered for 5 days. On day 5, the blood was collected from 10 rats through the superior satellite vein, and the liver and kidney were isolated for analysis. The rest of the rats were observed continuously for 15 days without any drug administrations. The rats were sacrificed under anaesthesia, blood was collected, and the kidneys and livers were isolated for examination.

#### Assessment of kidney function

The urine of the rats was collected using ZH-B6 metabolite cages (Anhui Zhenghua Biological Instrument Equipment Co., Ltd., Anhui, China). Blood and urine samples were collected for biochemical analysis, the kidneys and livers of rats were isolated and stored at −80 °C for expression analysis.

The quantity of urine collected after 24 h was determined, and the protein content of the urine was measured. Serum was isolated from rat blood for biochemical tests. Serum BUN (sBUN), serum Cre (sCre), sALB, and sTP were analyzed using a 7100 automatic biochemical analyzer (Hitachi, Japan), and urine Cre (uCre), mALB, and CSF were analyzed in the same manner as the serum index (Gong, He, Wang, Zou, et al. 2019). uTP = urine volume × CSF. uALB = urine volume × mALB. Cre clearance rate = (uCre × urine volume)/(sCre × 1440) (National Kidney Foundation 2002; Levey et al. 2020).

#### mRNA expression analysis

mRNA expression was analyzed using real-time PCR according to the references (He et al. 2019). Total RNA was extracted from the mouse hippocampus using a RNA extraction kit (Tiangen Biotech, Beijing, China) and reverse-transcribed to cDNA using the FastQuant RT Kit (Tiangen Biotech, Beijing, China) according to the manufacturer’s instructions. Real-time PCR analysis of specific genes was performed on a 7500 Real-Time PCR system (Applied Biosystems, USA) using SYBR Green master mix. The primers were designed by GenBank NCBI (https://www.ncbi.nlm.nih.gov/) and synthesized by Shenggong Biotechnology (Shanghai, China). The rat primers were as follows: CCAAT enhancer-binding protein (*Cebp*), sense: 5′-TACCGAGTAGGGGGAGCAAA-3′, anti-sense: 5′-AAGCAAGGGGCTAAGAACCC-3′; hepatocyte nuclear factor (*Hnf*), sense: 5′-GAGCTGCCAACCAAAAAGGG-3′, anti-sense: 5′-CCAGTTGTAGACACGCACCT-3′; nuclear factor Y (*Nfy*), sense: 5′-GATCGTTCAGACAGGAGCCA-3′, anti-sense: 5′-TGGCATTCACATACAACGGC-3′; and *Alb*, sense: 5′-GTGAGCGAGAAGGTCACCAA-3′, anti-sense: 5′-TTTCACCAGCTCAGCGAGAG-3′. β-actin served as an internal control (Hu et al. 2012).

#### Protein expression analysis

Protein expression was analyzed using Western blotting as previously described (Yu et al. 2019; Gong, Yan, et al. 2019). For Western blot analysis, primary antibodies against IL-6 (mouse monoclonal antibody, ab9324) and nephrin (rabbit monoclonal antibody, ab216341) were purchased from Abcam (UK), Anti-TGF-β1 (mouse monoclonal antibody, sc-130348) was purchased from Santa Cruz (USA), anti-podocin (rabbit polyclonal antibody, TA351459) was purchased from Origene (USA), anti-caspase-3 (rabbit polyclonal antibody, 9662S) was purchased from CST (USA), and anti-ALB (rabbit polyclonal antibody, 16475-1-AP) was purchased from Proteintech (USA). Goat anti-mouse IgG-HRP (ZB2305) and goat anti-rabbit (ZB2301) IgG-HRP secondary antibodies were purchased from ZSGB-Bio (Beijing, China). The targeted proteins were visualized with the Super Signal West Femto Chemiluminescent Substrate (Thermo Scientific Pierce), and the intensities of the visualized bands were analyzed using Quantity One software (Bio-Rad). β-Actin (mouse monoclonal antibody, TA-09, from ZSGB-Bio in Beijing, China) was used as an internal control. The data are expressed as the ratio to β-actin.

### Statistical analysis

All values are expressed as the mean ± SEM. The data were statistically analyzed using one-way analysis of variance (ANOVA) with F value determination. The *F*-test was performed using GraphPad Prism 8.01 software (GraphPad, San Diego, California, USA). Student’s *t*-test was performed between two groups after the *F*-test. *p*-Values below 0.05 were statistically significant. The statistical graphs were produced using GraphPad Prism 8.01 software as described above.

## Results

### DXM-induced increases in TP and ALB in normal and renal injury conditions

**Figure 1.**
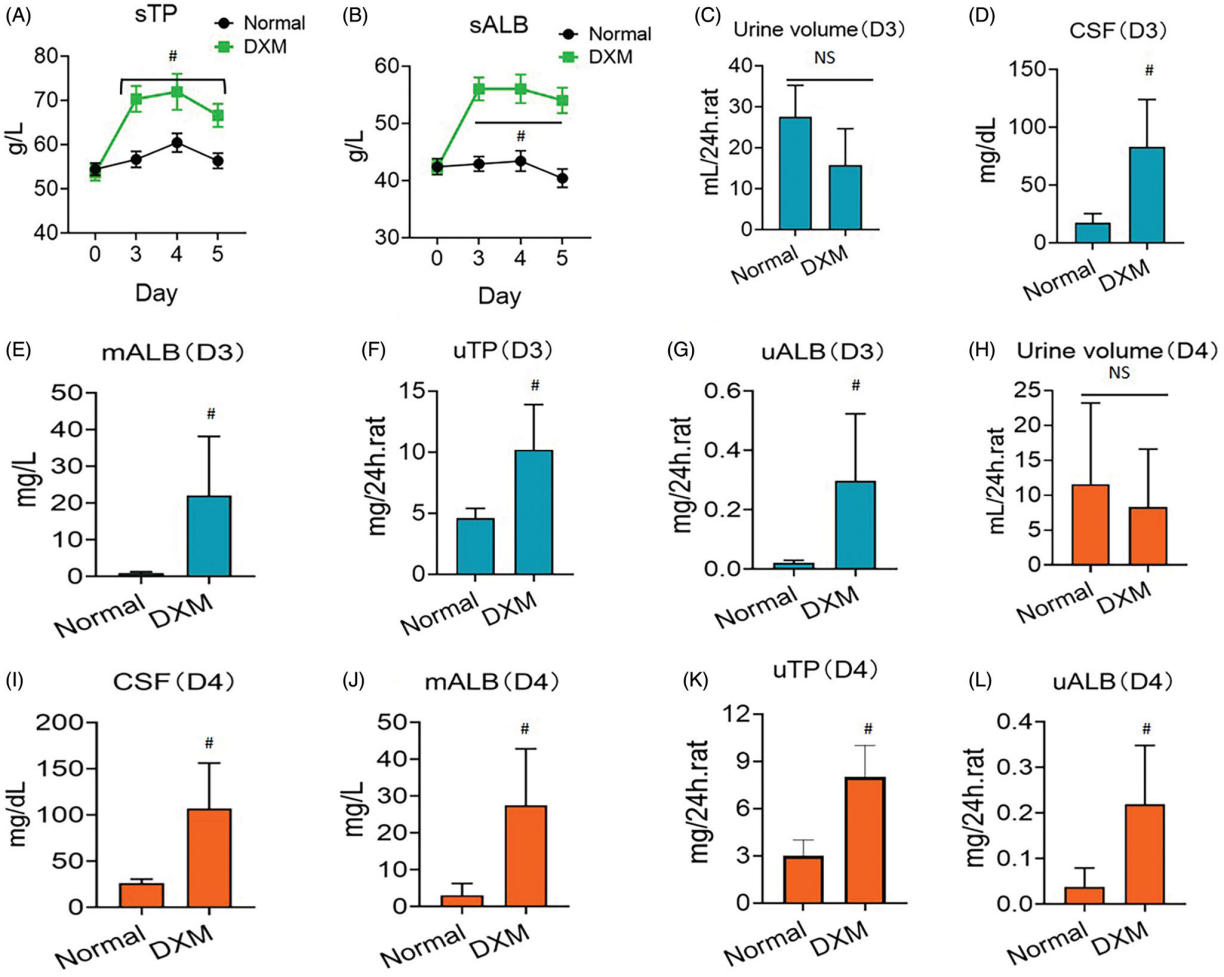
TP and ALB enhancement after dexamethasone (DXM) injection for 5 days in normal rats. (A, B) Serum TP and ALB. (C–G) Urinary proteins on day 3 (D3). (H–L) Urinary proteins on day 4 (D4). DXM was at 0.5 mg/kg by intramuscular injection. Data were expressed as mean ± SEM from nine rats in each group. #*p* < 0.05 vs. normal group. NS: no significance.

Figure 1 shows that DXM promoted ALB production in both serum and urine in normal rats. After 3 days of DXM administration, sTP and sALB both increased, peaking at day 4 and decreasing slightly on day 5 (Figure 1(A,B)). Although the 24 h urine level on day 3 was not different from that of the normal group, the concentrations of CSF and mALB were higher than those of the normal group, and uTP and uALB were both distinctly higher than those of the normal group (Figure 1(C–G)). Similar to day 3, uTP and uALB increased compared to those of the normal group on day 4 (Figure 1(H–L)). These results indicate that DXM can cause serum protein increases and abruptly increases urinary proteins.

**Figure 2.**
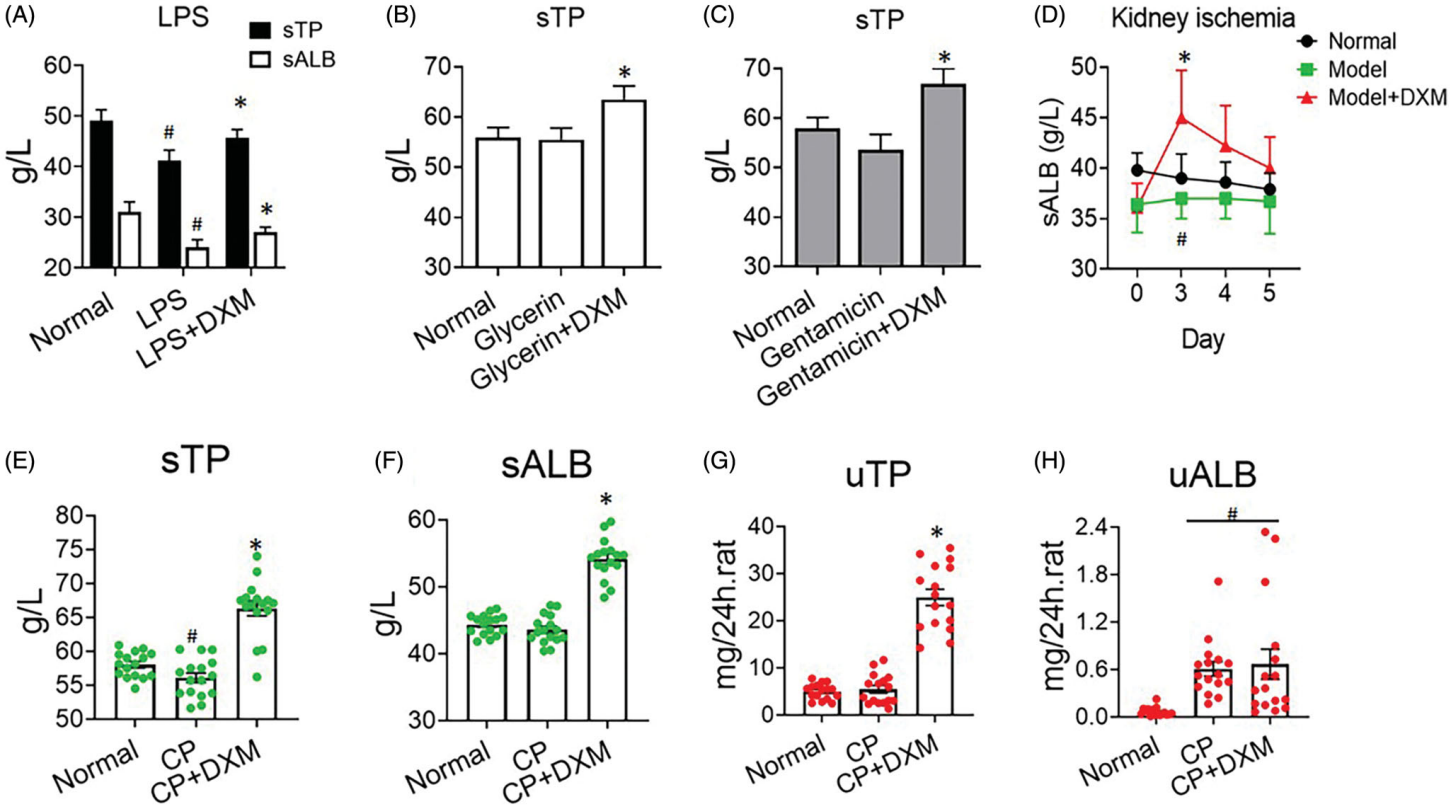
Production of ALB after 5 days of dexamethasone (DXM) administration during kidney injury by the agents. (A) Kidney injury induced by LPS. LPS: 10 mg/kg by introperitoneal injection. (B) Kidney injury induced by glycerine. Glycerine: 10 mL/kg (50% glycerine) by intramuscular injection. (C) Kidney injury induced by gentamicin. Gentamicin: 140 mg/kg by introperitoneal injection. (D) Kidney damage induced by the assay of renal ischaemia (30 min) and reperfusion (5 days). (E–H) Kidney injury induced by cisplatin (CP, 5 mg/kg intravenous administration). DXM was at 0.5 mg/kg by intramuscular injection. Data were expressed as mean ± SEM from 9 to 15 animals in each group. #*p* < 0.05 vs. normal group. *p < 0.05 vs. model groups (or LPS, Glycerine, Gentamicin, CP group).

In the acute renal injury experiment, sALB decreased after model induction with LPS, glycerine, gentamicin and renal ischaemia and reperfusion. However, the level of sALB was abruptly elevated after DXM administration (Figure 2(A–D)), suggesting that DXM could promote sTP and sALB, which was consistent with the normal conditions. In acute kidney injury induced by cisplatin, sTP and sALB were both decreased, and DXM antagonized this reduction (Figure 2(E,F)). Moreover, more uTP was secreted in the DXM groups than in the normal group (Figure 2(G)). However, CP could cause severe uALB increases, and DXM promoted a further increase in uALB (Figure 2(H)), suggesting that the glomerular basement membrane was injured even if DXM was administered. Thus, the results demonstrate that DXM can increase sTP and sALB and promote uTP and uALB in normal or injured conditions in the kidney.

### Dose- and time-dependent effects of DXM on TP and ALB under normal conditions

**Figure 3.**
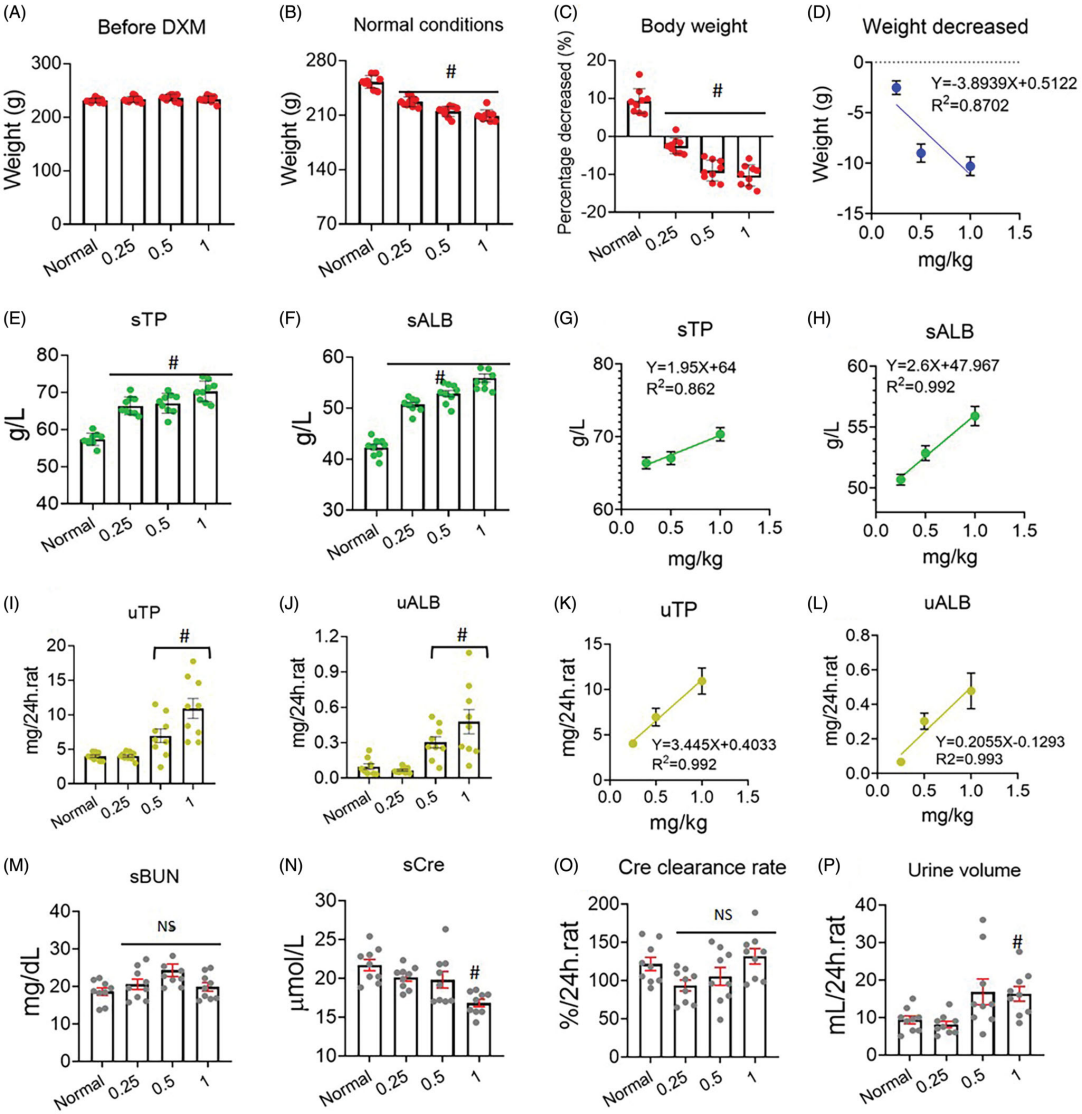
(A–P) Dose-dependent response of five day dexamethasone (DXM) injection on normal rat. DXM was at 0.25, 0.5 and 1 mg/kg by intramuscular injection. Data were expressed as mean ± SEM from nine rats in each group. #*p* < 0.05 vs. normal group. NS: no significance.

To examine the dose-dependent effect of DXM on serum protein production, we performed an experiment with DXM in normal rats. The results showed that DXM reduced rat body weight in a dose-dependent manner from 0.25 to 1 mg/kg (*R*^2^ = 0.8702, *p* < 0.05), suggesting that DXM influenced rats under physiological conditions (Figure 3(A–D)). Furthermore, sTP and sALB were both dose-dependently increased (R^2^_sT P_ = 0.862, *R*^2^_sALB_ = 0.992, *p* < 0.05) (Figure 3(E–H)), and uTP and uALB were both dose-dependently increased (*R*^2^_uTP_ = 0.992, *R*^2^_uALB_ = 0.993, *p* < 0.05) (Figure 3(I–L)). However, neither sBUN nor sCre was altered compared with those of normal rats (Figure 3(M,N)), and the clearance rate based on urinary Cre was not different from that of normal rats (Figure 3(O)), indicating that renal function was not damaged by DXM administration. Notably, the urine volume of the DXM groups was increased compared with those of the normal groups (*F* = 4.733, *p* = 0.0076), particularly in the medium (0.5 mg/kg) and high-dose groups (1 mg/kg) (*p* = 0.0534 and *p* = 0.0065, respectively) (Figure 3(P)), suggesting that DXM could promote urine release.

**Figure 4.**
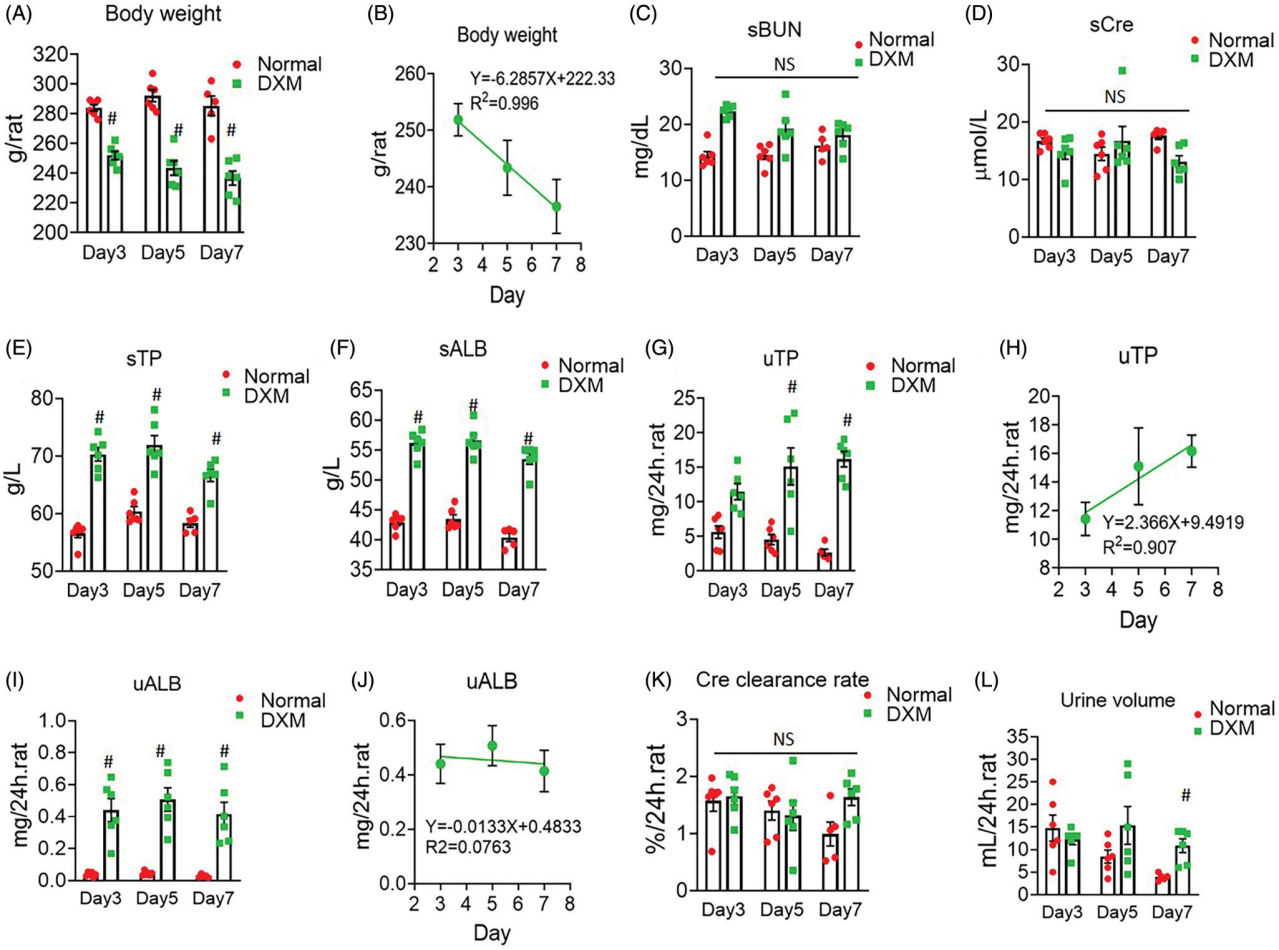
(A–L) Time-dependent response of dexamethasone (DXM) treatment on normal rats. DXM was at 0.5 mg/kg by intramuscular injection. Data were expressed as mean ± SEM from six rats in each group. #*p* < 0.05 vs. normal group. NS: no significance.

In addition to the dose-dependent effect of DXM on TP and ALB in normal rats, we conducted experiments to confirm the time-dependent effects of DXM. We administered DXM (0.5 mg/kg) for 3, 5 and 7 days and observed whether there were time-dependent changes. After DXM treatment in normal rats, the body weight decreased severely at 3, 5 and 7 days after DXM administration (*R*^2^ = 0.996, *p* < 0.05) (Figure 4(A,B)). sBUN and sCre were not different from those of normal rats (Figure 4(C,D)). However, sTP and sALB increased after 3 days of DXM administration, but there were no obvious further increases at day 5 and day 7 of DXM injection (Figure 4(E,F)), indicating no time-dependent effect. ALB in urine did not increase with time of DXM administration, which was the same as that of serum ALB. However, uTP did increase in a time-dependent manner after DXM injection (*R*^2^ = 0.907, *p* < 0.05) (Figure 4(G–J)). The Cre clearance rate did not differ from that of the normal groups, and the urine volume released on day 7 of DXM injection was not different from that of the normal group, but the difference was not time-dependent (Figure 4(K,L)). These results suggest that DXM can increase sTP and sALB abruptly on day 3 in a time-dependent manner and that day 3 is a critical time for the effect of DXM.

### Long-term impact of DXM on TP and ALB after ceasing treatment in normal conditions

**Figure 5.**
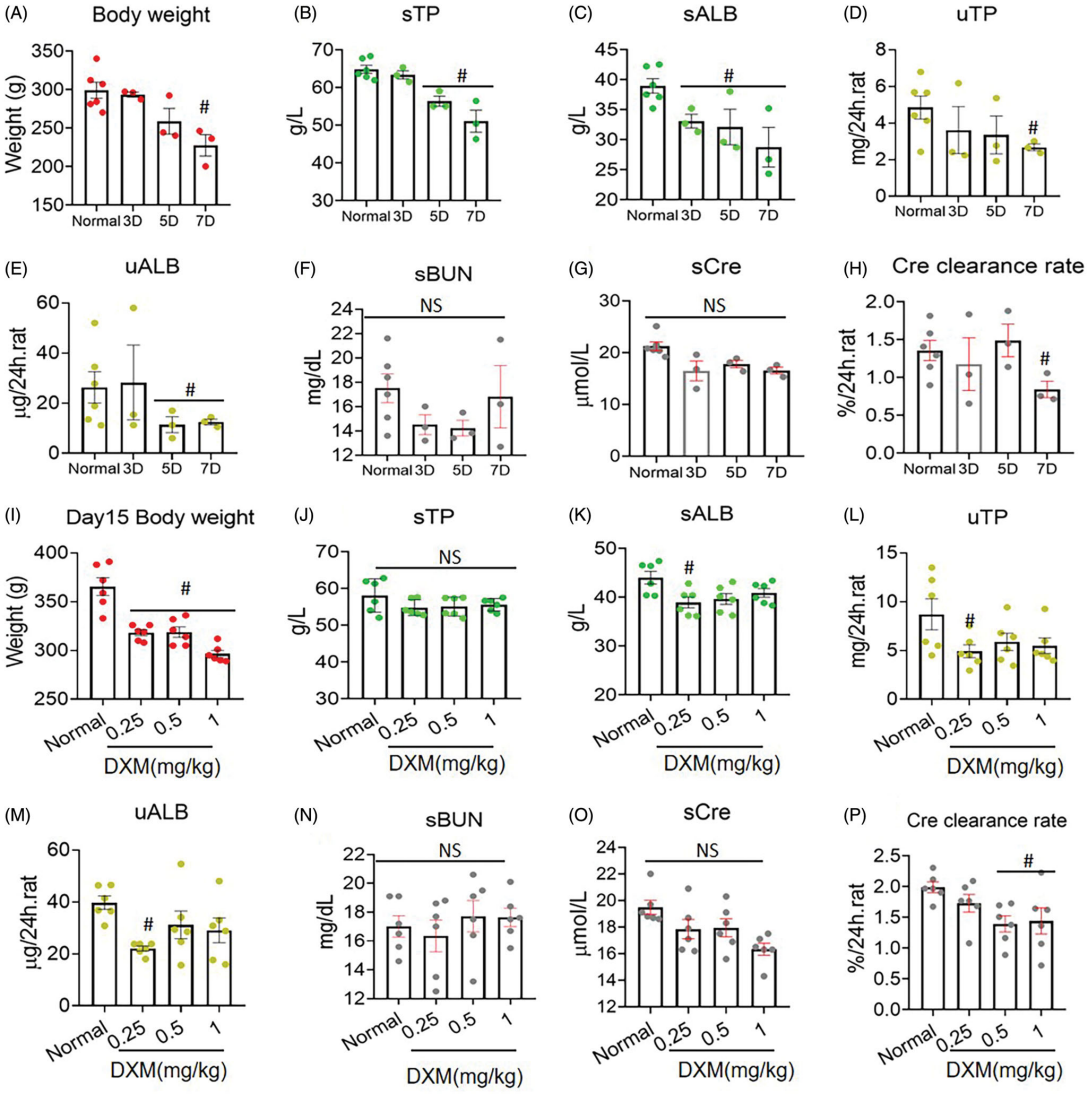
Kidney function changes at day15 after stopping dexamethasone (DXM) treatment in normal rats. (A–H) Different time for using DXM and stopping for 15 days. The dose of DXM was 0.5 mg/kg by intramuscular injection. (I–P) Different doses of DXM and stopping for 15 days. The doses of DXM were 0.25, 0.5 and 1 mg/kg by intramuscular injection. Data were expressed as mean ± SEM from three to six rats in each group. #*p* < 0.05 vs. normal group. NS: no significance.

To determine the long-term impact of these DXM-induced TP and ALB increases, we examined rat kidney function and other indices in the rats after 5 days of DXM treatment followed by 15 days without treatment (Figure 5). On day 15 after stopping DXM treatment, rat body weights were reduced in both dose- and time-dependent manners (Figure 5(A,I)), indicating that the dose- and time-dependent influences of DXM persist in rats without recovery even 15 days after cessation of DXM administration. However, sTP and sALB both decreased in response to different doses or treatment times (Figure 5(B,C,J,K)). uTP and uALB did not increase compared with those of the normal group (Figure 5(D,E,L,M)), and sBUN and sCre were not higher than those of the normal group (Figure 5(F,G,N,O)). Notably, the clearance rate of Cre was not different from that of the normal group, suggesting that renal function was not worse than that of the normal after halting DXM stopped administration for 15 days.

### Dose-dependent effect of DXM on TP and ALB in cisplatin-induced renal damage

**Figure 6.**
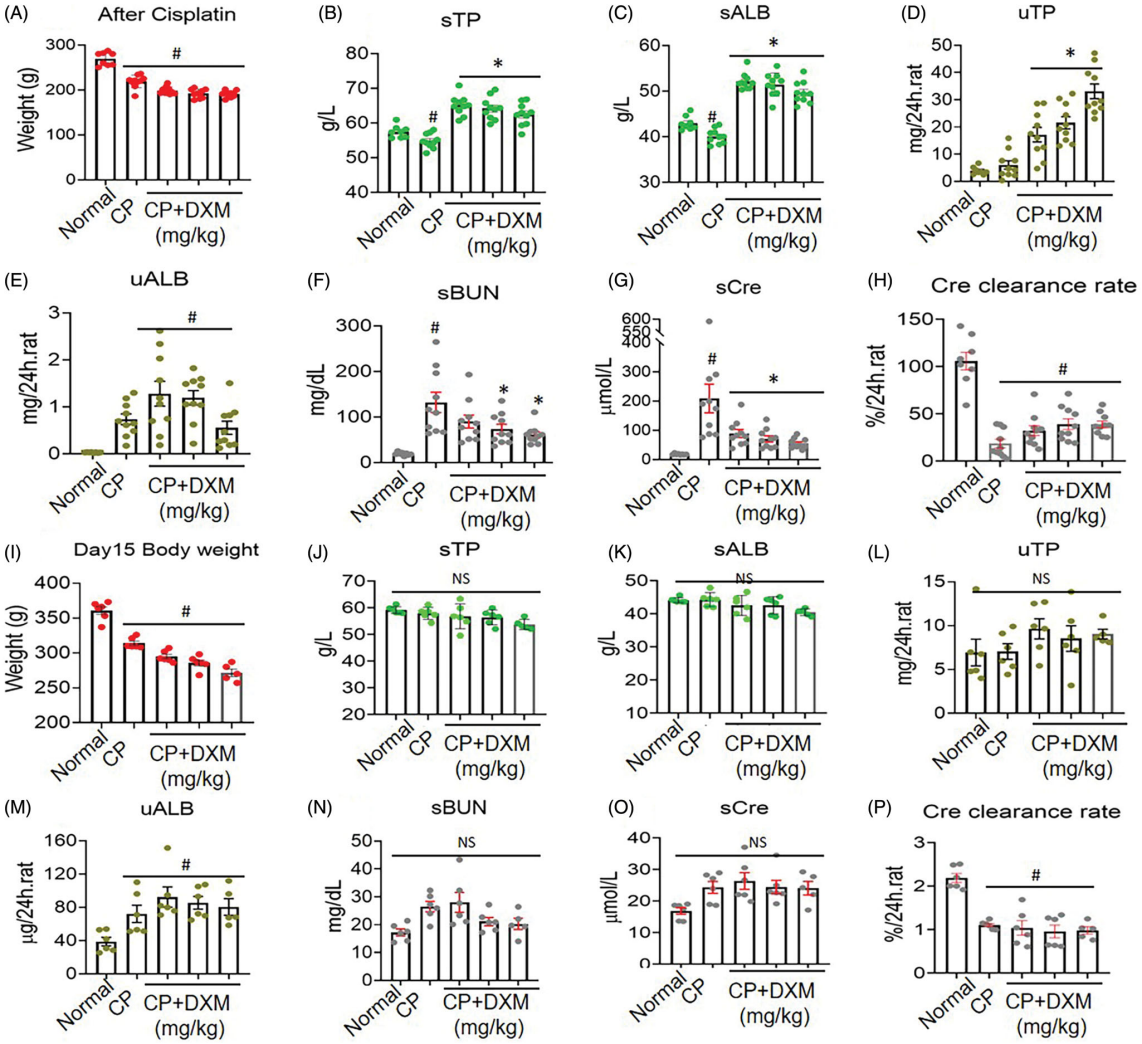
Five day dexamethasone (DXM) treatment with different doses on the kidney damage induced by cisplatin (CP). (A–H) Changes of kidney just after 5 days of DXM injection. (I–P) Changes of kidney after 15 days of DXM stopping injection. The dosages of DXM were 0.25, 0.5 and 1 mg/kg by intramuscular injection. CP was at 5 mg/kg intravenous administration. Data were expressed as mean ± SEM from 6 to 10 rats in each group. #*p* < 0.05 vs. normal group. *p < 0.05 vs. CP group. NS: no significance.

To determine the effect of DXM on protein production in pathological conditions, we conducted an experiment on the cisplatin-induced acute renal injury. In the experiment, cisplatin caused declines in the weights of rats, and sTP and sALB decreased (Figure 6(A,B)). After 5 days of DXM treatment in rats with cisplatin-induced kidney damage, sTP and sALB increased distinctly compared with those of CP and normal group rats, and uTP and uALB were clearly increased, suggesting that DXM dose-dependently promoted protein production in the context of cisplatin-induced damage to the glomerular membrane (Figure 6(B–E)). DXM could antagonize cisplatin-induced serum BUN and Cre increases, and the Cre clearance rate in the large dose (1 mg/kg) groups were improved, clearly suggesting that rat kidney function was mildly improved (Figure 6(F–H)). At 15 days after stopping DXM, the body weight in the CP + DXM groups and CP groups decreased (Figure 6(I)). However, sTP and sALB in the CP + DXM and CP groups showed no differences compared with those of the normal group, indicating that 15 days later, sTP and sALB had decreased to normal (Figure 6(J,K)). Urinary ALB in the CP + DXM groups remained at a high level compared with that of the normal group, and uTP in the high-dose DXM groups was higher than that of the normal group (Figure 6(L,M)). Moreover, uALB in the CP groups was also increased, suggesting that the damage induced by cisplatin persisted. Serum BUN and sCre remained increased in the low-dose CP + DXM groups; however, sBUN in the medium- and high-dose groups declined to normal levels, but there was no effect on sCre (Figure 6(N,O)). Similar to that of the CP groups, the Cre clearance rate in the CP + DXM groups clearly declined (Figure 6(P)), indicating sustained damage to renal function in rats.

### Effect of DXM on mRNA and protein expression

**Figure 7.**
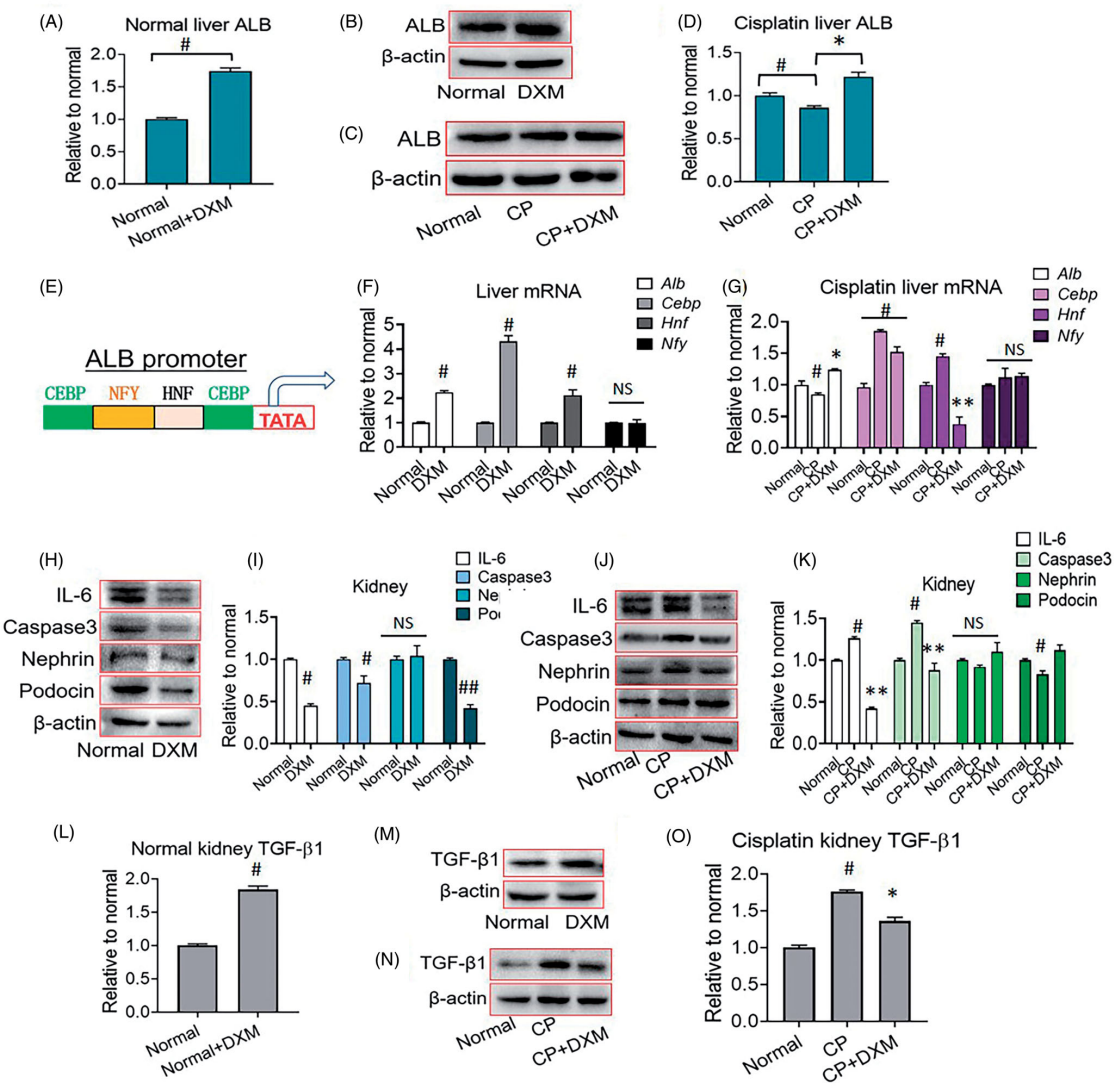
Protein and mRNA expression of rat liver and kidney after dexamethasone (DXM) injection. (A–D) ALB expression in rat liver. (E) Schematic view of ALB promoter. (F) mRNA expression of the enhancers in ALB gene transcription start site in normal rat liver. (G) mRNA expression of the enhancers in ALB gene transcription start site in rat liver after cisplatin (CP) injection. (H, I) Protein expression of normal rat kidney. (J, K) Protein expression of injury kidney induced by CP. (L–O) The protein expression of TGF-β1 in rat kidney 15 days after stopping DXM. Data were expressed as mean ± SEM from six to ten rats in each group. #*p* < 0.05, ##*p* < 0.01 vs. normal group. *p < 0.05, **p < 0.01 vs. CP group. NS: no significance.

ALB synthesis mainly occurs in the liver. We hypothesized that DXM could promote ALB production in the liver. Thus, we measured ALB protein expression, and the results showed that ALB protein expression was upregulated in the DXM groups compared with the normal group (Figure 7(A–D)). Thereafter, we analyzed the reason that DXM enhanced liver ALB expression. Figure 7(E) shows that DXM could increase the mRNA expression of *Alb, Cebp* and *Hnf*, the enhancer at the gene transcription start site of *Alb*, indicating that the mechanism by which DXM affects the gene transcription of *Alb* involves two enhancers, *Cebp* and *Hnf*. However, in the cisplatin-induced renal damage rat model, DXM affected *Cebp* but not *Hnf*, suggesting a difference between normal and pathological conditions, which needs to be studied further. DXM had no effect on the enhancer *Nfy* under either normal or cisplatin-induced conditions (Figure 7(F,G)). Figure 7(H–K) shows the inflammation in the glomerulus of the kidney in both normal and pathological conditions. Under normal conditions, DXM could suppress the protein expression of IL-6 and caspase-3, showing anti-inflammatory and anti-apoptotic effects. Moreover, DXM downregulated the protein expression of podocin, a key cytokine in the renal glomerular basement membrane, suggesting that DXM could damage glomerular filtration (Figure 7(H,I)). After cisplatin injury, IL-6 and caspase-3 were upregulated, indicating damage to the rat kidney, and DXM antagonized this upregulation, protecting the kidney from cisplatin-induced damage. In addition, cisplatin could suppress nephrin and podocin expression, and DXM antagonized this suppression and ameliorated the expression, which was beneficial for the renal glomerular basement membrane because nephrin and podocin affect the podocyte slit diaphragm (Figure 7(J,K)). After 15 days without DXM, TGF-β1 in the DXM groups was apparently upregulated under normal conditions and downregulated mildly under pathological conditions to antagonize the increase caused by CP. This discrepancy, similar to that of podocin expression, brings us a new and interesting perspective that needs to be studied further.

## Discussion

DXM is commonly used clinically to treat kidney injury through its anti-inflammatory and renoprotective effects. However, our results show that DXM can promote the synthesis of ALB in the liver, increase sALB and sTP, and simultaneously promote the excretion of urine proteins due to its effects on membrane permeability and anti-inflammatory effects. Moreover, the protein increases caused by DXM produced a long-term effect on renal function after drug withdrawal. Further studies explored whether the activation of the enhancers Hnf and Cebp in the ALB gene transcription start site contributed to the underlying mechanism by which DXM promoted ALB expression in hepatocytes, as well as the increase in sALB.

Podocytes are an important part of the permeability of the glomerular basement membrane, and during DXM use, podocytes are seriously damaged, resulting in glomerular injury (Jefferson et al. 2011; Campbell and Tumlin 2018). ALB is mainly absorbed in the renal proximal tubules (Russo et al. 2007). Heavy excretion of ALB can decrease ALB reabsorption and damage epithelial cells in the tubules. DXM promotes leakage of glomerular filtration membranes (Martins et al. 1998; Fleck 1999). This leakage increases the rate of ALB leakage into the serum and thus increases urinary ALB and TP excretion. In addition, elevated serum ALB inevitably causes injury to the glomerular filtration membrane, damaging podocytes. Thus, we need to pay attention when using DXM. Whether this effect can further exacerbate the original kidney injury is worth further study. Long-term treatment with DXM can cause damage to glomerular podocytes and endothelial cells, resulting in mild mesangial expansion, segmental or global hyalinosis/sclerosis in deep cortical glomeruli, and focal tubular changes. The effect of glomerular hyperfiltration may be only partially involved in the pathogenesis of this dexamethasone-induced glomerulopathy (Chen et al. 1998).

The use of DXM in normal rats significantly increased sTP, sALB, and protein concentration in the urine. Moreover, renal podocin expression was reduced, indicating that DXM could promote ALB synthesis and consequently influence glomerular basement membrane by reducing podocin. These protein alterations by DXM occurred in both dose- and time-dependent manners. In addition to the body, weight decreases at 15 days after stopping DXM, the Cre clearance rate (reflecting renal glomerular filtration) declined in response to medium and high doses (0.5 and 1 mg/kg) and long-term administration (7 days) of DXM (Figure 5(H,P)), which needs to be further studied.

There are three enhancer elements in the *Alb* gene transcription start site, *Cebp*, *Hnf* and *Nfy* (Lichtsteiner and Schibler 1989; Vorachek et al. 2000). To determine the underlying mechanism by which DXM promotes ALB excretion, we examine DXM and the enhancers. Our results showed that DXM could upregulate the mRNA expression of *Cebp* and *Hnf* without affecting *Nfy*, suggesting a mechanism that correlates with *Cebp* and *Hnf* expression, strengthening *Alb* expression.

To determine the effect of DXM on ALB during renal injury, we studied the effect of DXM on ALB alterations in the context of renal damage induced by CP. After CP injection, sTP and sALB were decreased, uALB was distinctively excreted, and sBUN and sCre were increased, indicating that renal function was damaged. DXM obviously increased sTP and sALB and the excretion of uTP and uALB without any dose-dependent effects. DXM decreased sBUN and sCre and mildly increased the Cre clearance rate, suggesting that DXM antagonized CP-induced renal damage. Notably, at 15 days after stopping DXM, uALB remained at a high level, and the Cre clearance rate remained at a lower level than that of the normal control, suggesting that DXM failed to antagonize CP injury at that time. Thus, we might conclude that DXM does not completely restore kidney function during acute injury and that a high ALB level does not facilitate improvements in kidney function.

The transmembrane proteins nephrin and podocin are essential components of the glomerular basement membrane complex. Nephrin is a key component of the podocyte slit diaphragm. The slit diaphragm is the cell–cell junction between adjacent podocyte foot processes and is part of a large multiprotein complex that is involved in maintaining podocyte integrity and in signalling to the podocyte actin cytoskeleton (Welsh and Saleem 2011; Perico et al. 2016). Nephrin forms zipper-like interactions to maintain the structure of the podocyte foot processes and nephrin-induced signalling is greatly enhanced by podocin, which binds to the cytoplasmic tail of nephrin (Huber et al. 2001). Thus, podocin can activate nephrin signalling and in turn disturb the gap between the podocytes, as well as the permeability of the glomerular basement membrane (Brinkkoetter et al. 2013; Saleem 2019). Downregulation of podocin could increase the permeability of the glomerular basement membrane, causing proteinuria.

Our results showed that DXM promoted uALB excretion and downregulated podocin expression in normal rats, indicating that this excretion was correlated with the decrease in podocin (Figures 3(G,I) and 7(I)). However, in CP-induced renal damage rats, DXM promoted podocin expression, but uTP and uALB levels remained high (Figures 6(D,E) and 7(K)), which requires further study. Another interesting finding was that DXM could induce TGF-β1 upregulation on day 15 after stopping DXM in normal rats, but TGF-β1 was downregulated by DXM in the kidneys of rats with CP-induced kidney damage, indicating a discrepancy in TGF-β1 expression in normal and pathological conditions. TGF-β1 is highly correlated with renal fibrosis in chronic kidney disease (Ma and Meng 2019; Yang et al. 2020); therefore, such robust upregulation of TGF-β1 in normal rats suggested that DXM induced kidney fibrosis in the long term. Moreover, the effects of DXM against CP-induced TGF-β1 upregulation provide ideas for further research.

It has long been observed that DXM can increase uALB in normal animals (Knepper et al. 1975; Zager 1981; Chen et al. 1998). Our results show that DXM has a dose-effect relationship with ALB synthesis. How to maintain this anti-inflammatory effect while reducing the minimum ALB synthesis is an important aspect of kidney injury treatment. Our results showed that when DXM was below 0.25 mg/kg, it had no significant effect on either sALB or uALB while maintaining its anti-inflammatory effect, which should be considered clinically.

## Conclusions

We comprehensively investigated the effects of DXM on protein production in normal and injured conditions in the kidney. DXM can act on the enhancers Cebp and Hnf in the gene transcription start site of ALB and promote ALB production. High levels of secreted ALB cause proteinuria. In acute kidney injury, DXM causes high blood ALB levels and proteinuria and has a long-term effect on renal glomerular filtration. Thus, caution should be used when administering DXM in cases with a risk of kidney impairment.

## Authors’ contributions

Jun Li, Lijun Du and Yulin Feng conceptualized the study. Qin Gong, Jilei Yin, Yingying Luo, Jun Li and Lijun Du designed the experiments; Qin Gong, Jilei Yin, Mulan Wang, Lingling He and Fan Lei performed the experiments, Qin Gong, Shilin Yang and Lijun Du analyzed the experimental data; Qin Gong, Jilei Yin, Jun Li, Yulin Feng and Lijun Du wrote the paper. All authors read and approved the final manuscript.
